# Perceptions of Smoking Stigma Among African Americans: A Qualitative Study

**DOI:** 10.1093/ntr/ntae127

**Published:** 2024-07-16

**Authors:** Denine R Crittendon, Alison C Brecher, Samantha Okere, Richard Hass, Rosemary Frasso, Rickie Brawer, Charnita Zeigler-Johnson

**Affiliations:** College of Population Health, Thomas Jefferson University, Philadelphia, PA, USA; Fox Chase Cancer Center, Philadelphia, PA, USA; Sidney Kimmel College of Medicine, Thomas Jefferson University, Philadelphia, PA, USA; College of Population Health, Thomas Jefferson University, Philadelphia, PA, USA; College of Population Health, Thomas Jefferson University, Philadelphia, PA, USA; College of Population Health, Thomas Jefferson University, Philadelphia, PA, USA; College of Population Health, Thomas Jefferson University, Philadelphia, PA, USA; Fox Chase Cancer Center, Philadelphia, PA, USA

## Abstract

**Introduction:**

African Americans/Blacks (AAB) are at increased risk for morbidity and mortality from smoking-related diseases including lung cancer (LC). Smoking stigma is believed to be a primary barrier to health care-seeking for people who smoke. Previous studies illustrate that perceptions of smoking vary across populations. However, little is known about the prevalence of smoking stigmas among AAB. The purpose of this study was to increase understanding of the perception of cigarette smoking by AAB.

**Aims and Methods:**

We conducted free-listing interviews in which individuals listed all-thoughts and feelings regarding smoking and health-related questions with a convenience sample of eligible AAB adults (*n* = 58) in the Philadelphia region. Additionally, we collected participant self-reported demographic data. Data were cleaned and the salience of each term was computed using Anthropac. Graphical methods were then used to determine salient responses across groups stratified by gender, age, education, and smoking status.

**Results:**

The sample had a median age of 51 years and was 67.2% female. Most participants had completed college (58.6%) and had never smoked (74.1%). Regarding their perceptions of people who smoke cigarettes, results showed that “smelly,” “health hazard,” and “judgment” were the most salient terms among all-participants. Overall, “smelly” and “unhealthy” were salient for both males and females. However, “dental,” “dirty,” “addictive,” and “habit” were also salient among males. Phrases such as “unhealthy” and “addictive” were primarily salient for older participants (>51 years) versus “smelly” for younger participants. The term “smelly” was salient among all-education levels. However, “unhealthy” was also salient among those with less than a 4-year college degree. Moreover, the terms “smelly” and “annoying” were most common among people who smoke as opposed to “health hazard” among people who don’t smoke.

**Conclusions:**

We observed that the most stigmatizing language was primarily associated with perceptions of negative social interactions, social judgment, and health-related concerns. Future studies are needed to explore how smoking-related stigmas impact patient adherence to smoking cessation programs and LC screening protocols.

**Implications:**

Little is known about the prevalence of smoking stigmas among AAB. This study explores the AAB perspective of cigarette smoking and related stigmas. Among AAB, smoking is represented by stigmatizing language across gender, age groups, and smoking history. It is primarily associated with negative social interactions, social judgement, and health-related concerns indicating that smoking stigma is a concern for AAB individuals who smoke. Further research is warranted.

## Introduction

Cigarette smoking is the leading cause of preventable disease, disability, and death in the United States, accounting for approximately one in five deaths in the nation.^1^ In 2021, there were an estimated 28.3 million adults in the United States who currently smoked cigarettes (defined as those smoking at least 100 cigarettes during their lifetime and who currently smoke), with more than 16 million Americans living with smoking-related disease.^1^ There are demographic disparities regarding smoking prevalence based on age, race/ethnicity, and education level. Current data finds that smoking is highest in those aged 25–44 and 45–64 years old.^1^ Moreover, current cigarette smoking was highest among adults racialized as American Indian/Alaska Native and as Multiracial compared to adults of Asian descent.^2^ Concerning commercial tobacco, adults racialized as African American/Black (AAB) begin smoking at older ages than adults racialized as White but are more likely to experience smoking-related complications and death compared to other racialized groups.^1^ Also, cigarette smoking is highest among persons with lower educational attainment compared to persons with a college and/or graduate degree.^1,2^ While cigarette smoking remains a leading cause of preventable morbidity and mortality in the United States, long-term cigarette use has decreased over the last 30 years among adults and youth.^2^ The proportion of individuals smoking more than 15 cigarettes per day has also decreased.^2^

In line with declining cigarette use, the perception of cigarette smoking has shifted away from being socially acceptable.^3,4^ This is evidenced by smoke-free laws,^5^ tobacco-free hiring processes,^6^ and the addition of tobacco surcharges to health insurance premiums.^7^ Compared to people who smoke, people who do not smoke tend to hold negative perceptions of smoking behavior and prefer to limit interactions with people who smoke.^8^ These changes in social (or perhaps public) acceptability also coincided with increasing stigma against people who smoke and smoking behavior.^9^

Stigma is an attribute considered undesirable and unpleasant by society differentiating the person from other members of the community.^10^ Smoking stigma fits this definition.^11^ Several decades ago, smoking was viewed as “cool” and “mysterious”^12,13^; however, the social status regarding people who smoke has diminished in recent decades.^14^ Smoking stigma has been measured in the past using qualitative interviews that identified participants’ views on perceived (public) stigma and internalized (self) stigma.^15^ Public stigma is the perception held by others that the individual affected by a certain condition (eg nicotine dependence) is socially undesirable.^16^ People who smoke may internalize this public stigma and/or create negative feelings about themselves, thereby generating self-stigma.^16^

Stigma is a multidimensional and potentially multi-level barrier to disease prevention and health care outcomes, including lung cancer (LC) screening and treatment.^17^ There are societal and structural biases and stigmatization of people who smoke (including in health care). There are also perceived and internalized stigmas that hinder health care-seeking behavior among people who smoke. Stigma affects the psychosocial, communication, and behavioral outcomes across the health care continuum.^17^ The role of patient-reported stigma in limited engagement and adherence to preventive care and treatment is significant.^17,18^ Stigma has led to delays in health-seeking behaviors which may, in turn, result in delays in diagnosis.^19^ Not only do individuals with stigmatized conditions anticipate experiencing social stigma,^20^ but also individuals with stigmatized conditions experience stigma in health care settings.^21^ Stigma is a complex, global phenomenon that can negatively impact the health and well-being of people living with multiple health conditions viewed as contagious, dangerous or incurable. Such conditions include HIV, poor mental health, epilepsy, and cancer, with effects on care, treatment, and quality of life.^22^

Despite cigarette smoking perceptions being mostly negative, perceptions vary by demographics and smoking status. For example, there have been mixed opinions about which smoking behaviors are most harmful. In one study, the majority of patients who were recently diagnosed with cancer believed that continued smoking after cancer diagnosis affected quality of life, survival, and fatigue. However, these findings varied by smoking status, as people who currently smoke were less likely to perceive continued smoking as harmful compared to people who never smoked or people who formerly smoked.^23^ In addition, those who had a more significant smoking history were less likely to believe that smoking was detrimental to their health than their counterparts. Amrock and Weitzman^24^ found that adolescents reported beliefs of heavier smoking patterns being harmful, however, they were less likely to report this to be the case with lighter, intermittent smoking. In this same study, those who had light or intermittent smoking patterns, those who used other tobacco products, or those who had a family member who used tobacco were also less likely to view their smoking patterns as negative practices. Individuals who deny their current smoking status also are least likely to consider preventive behaviors, such as smoking cessation or reduction.^9^

Perceptions of cigarette smoking-related stigma also vary where multiple identities are concerned. Stuber et al.^14,25^ examined how policies (eg smoke-free air laws) and social factors (eg social norms) contributed to the formation of smoking-related stigma among study respondents of various racial or ethnic backgrounds who had a history of smoking. The researchers determined that persons racialized as White perceived more smoking-related stigma than persons racialized as AAB or identified as Hispanic in ethnicity. The authors believed this was possibly attributable to a “segregation effect,” meaning that tobacco control policies may be less strictly enforced in neighborhoods where racialized as AAB or identified as Hispanic are highly concentrated. Moreover, the study suggested Blacks and Latinos may be more likely to hold perceptions of stigma and discrimination that are related to their racial and ethnic identities with a tendency to disregard smoking-related stigma. Lipperman-Keda et al.^26^ investigated the perceptions of smoking-related stigma among persons racialized as African American or persons of Hispanic ethnicity who identify as a sexual and gender minority (SGM) as influenced by the intersection of multiple identities. These identities include those related to race, class, or vulnerability due to living in low-resourced socioeconomic conditions. Findings showed that persons racialized as African American who identify as a SGM and who also live in low socioeconomic conditions are most likely to perceive smoking-related stigma, experience differential treatment because of their smoking, and state they socially withdraw from people who don’t smoke. Yet, they are less likely to conceal their smoking status.

Although both studies explored the perceptions of smoking-related stigma among populations racialized as Black/African American, and/or the origins of perceptions formation, the studies were focused on policy and social norms; or the intersection of race, sex and gender identity, and socioeconomic status as they primarily related issues of public stigma rather than self-stigma among people with a smoking history. For instance, the studies are centered on public stigma in the context of discriminatory structural practices such as barring people who smoke from being employed, differential treatment experienced by people with a smoking history in social settings, disparate views on smoking that are observed among racialized populations, and behaviors exhibited by people who feel compelled to hide their smoking habits. Although Lipperman-Keda et al.^26^ briefly mention that people may conceal their smoking history from health care providers to avoid judgment or negative patient-provider encounters, none of the studies specifically explore self-stigma and its relationship with preventive health care-seeking behavior among patients racialized as Black/African American. They also did not include a growing population of people who never smoked cigarettes. In our study, we included a sample with diverse smoking histories to address both public and self-stigmas among people with diverse smoking histories and how the data may potentially support initiatives to remove barriers to care such as lung cancer screenings or smoking cessation programs.

AAB is a population with a unique susceptibility to smoking-related disease and a high risk for poor health outcomes. The lower threshold for developing lung cancer is likely multifactorial, including social factors and racism.^27^ This population also perceives a greater risk of disease from smoking than other race groups.^28^ Vulnerable populations may be at greater risk of experiencing stigma, which may impact their success at quitting. While stigma has moved some individuals to attempt smoking cessation after experiencing antismoking announcements from the public,^29^ stigmatization has also generated emotional, psychological, cognitive, and attitudinal reactions that impede smoking cessation.^30^ With such contrasting results regarding stigma on smoking cessation, further analysis on how stigma arises in vulnerable populations is warranted. Despite the increased perception of the risk posed by smoking, AAB who smoke may face a double stigma by being part of a racial/ethnic minority group confronting other prejudices and discrimination.^31^ The double stigma can impede treatment and hinder prevention tactics beyond what is observed for other populations.^31^

This study contributes to the literature by updating current perceptions of smoking and nonsmoking behaviors among AABs. Free listed perceptions of AAB by smoking history (including people who never smoked) are a novel elicitation study design component. The study examined how the concept of smoking stigma manifests in AAB populations while looking into its prevalence in this population and which demographic groups are more likely to report various types of stigmas (eg social stigma and perceived stigma).

## Materials and Methods

The aim of this study was to describe perceptions of cigarette smoking and health among community members who identify as AAB. To investigate the conceptualization of cigarette smoking by AAB, our study team conducted a qualitative study utilizing free listing as a means of data collection. This interviewing method was selected because it is appropriate for exploring what comes to mind when a participant is asked to think about a specific topic.^32^ Free listing is seen as a particularly useful technique of engagement for populations identified as vulnerable, marginalized and historically traumatized. It centers community “voice” in academic research, considers how a concept like a stigma may frame perceptions related to racialized social position and smoking status and allows for the exploration and comparison of communication nuances that surround meaning and experience across different populations^32,33^

### Population Inclusion Criteria

Eligible participants for the study included persons who self-identified as AAB, resided in the Philadelphia, Pennsylvania region, were at least 18 years of age, regardless of smoking history, and who visited a primary care doctor at least once since being an adult. The Thomas Jefferson University Institutional Review Board (Control # 21E.250) approved the project and all-participants verbally consented prior to taking part in the study.

### Pilot Testing

After initial team development of demographic and free-listing questions, we initiated a pilot study. Two members of the research team (C.Z.J. and D.C.) tested the questions with a convenience sample of eligible participants (*n* = 4) who were excluded from the main study. Based on the feedback received, a finalized draft of the demographic and free-listing questions was completed and the study team moved forward with data collection.

### Data Collection

A convenience sample of participants (*n* = 58) who resided in the Philadelphia, Pennsylvania region between July and August 2021 was recruited by two AAB members of the research team (C.Z.J. and D.C.) using a snowball technique. Saturation in free listing could be achieved in homogeneous populations with a minimum of 20 interviews.^32^ The research team decided the researchers (C.Z.J. and D.C.) would perform a minimum of 25 interviews each.

The consent process and free-listing interviews were completed either by phone, via Zoom, or in-person in ~10 min each by two AAB research team members. First, demographic data (Table 1) were recorded at the beginning of each interview. Two questions were included in the survey to assess the smoking status of participants. They were: (1) Have you smoked at least 100 cigarettes in your entire life? and (2) Do you now smoke cigarettes every day, some days, or not at all?After completing the survey, each participant responded to four prompts: “List all the things that come to mind when you think about…” 1. “A person who smokes cigarettes,” 2. “Secondhand smoke,” 3. “How people treat people who smoke cigarettes,” and 4. “Talking to your doctor about your health.” The question about talking to the doctor about health was presented more generally (and less specifically about smoking) because we included people who have no smoking history. The results capture different dimensions of stigma including public stigma and perceived stigma in the case of those who smoke.

**Table 1.** Demographics for the Sample of African-American Community Members in Southeastern Pennsylvania: Smoking Stigma Free-Listing Study (*N* = 58)

Demographic		Sample Size *N*	Percent
Gender	Female	39	67.24
	Male	19	32.76
Education attainment	<4 y of college	24	41.38
	≥4-y college	34	58.62
Smoking status	Never	43	74.14
	Ever	15	25.86
Age group	<51 y	29	50.00
	≥51 y	29	50.00

Data were entered into Excel. Each researcher transcribed the collected data into five separate Excel sheets. One sheet housed demographic data while four sheets contained the participant responses to each of the four questions asked during scheduled interviews. At the end of the interviewing period, the 10 separate sheets were cleaned and merged. The Consolidated Criteria for Reporting Qualitative Research (COREQ) Checklist was used to document items included in our qualitative study.^34^

### Data Cleaning and Merger

Free-listing responses from different participants are often variations of the same general category (eg “smells bad,” and “odor”) and must be cleaned prior to salience analysis. Three researchers (S.O., C.Z.J., and D.C.) collaborated to perform data cleaning of the free-listing responses. During the initial meeting, a key was created to store codes that were developed to collapse word synonyms and words having similar meanings into categories. The codes were comprised of words actually spoken and recorded during interviews. For instance, the code “Bad Breath” was a category created to capture when participants stated words or phrases such as “bad breath,” “cigarette breath,” “stinky breath,” or “bad taste in mouth.” Repeated iterations of this process occurred over multiple sessions until each of the four sets of responses was cleaned (one for each prompt.) Two researchers worked as a team to ensure data accuracy. A fourth researcher (RF) was consulted to settle any disagreements encountered concerning code categories. Once data cleaning was complete, all-coded data was merged into one Excel sheet.

## Data Analysis

**Figure 1.**
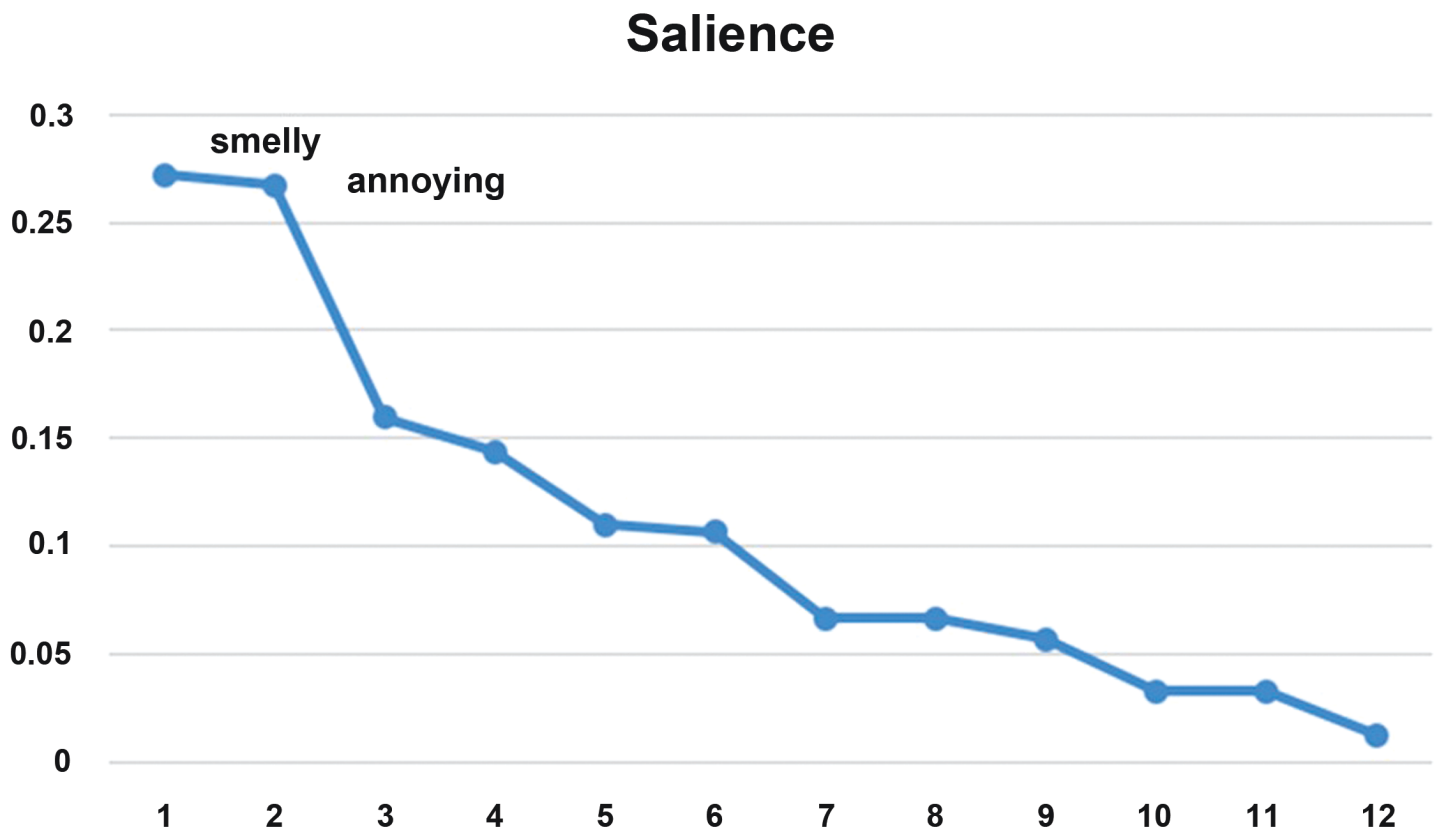
Example of a scree plot.

The primary goal of the study was to identify terms concerning smoking stigma that are most salient among AAB communities. To achieve this goal, the merged Excel sheet was converted to a text file, and then entered into Anthropac, a software program that performs analysis of free-listing data.^32^ The key metric computed by Anthropac is Smith’s *S*, defined as:


$$\begin{array}{l} S=\frac{1}{N}\underset{ij}{\sum }\frac{L_{i}-R_{j}+1}{L_{i}} \end{array}$$

where *L* is the length of each list, *Rj* is the rank of item J in the list, and *N* is the number of lists in the sample. The sum is then over all-lists *i* containing term *j*.^35^ Smith’s *S* is essentially a weighted average rank (order in a particular list) for each term with larger values indicating a term tends to be first in many lists. Demographic data collected from participants were used to stratify results according to gender, age, education, and smoking status. STATA/SE 13.1 was used to analyze the demographic data.^36^ To identify the most salient terms, a scree plot was created for each list and all-strata. Terms that fall above and to the left of a pronounced “elbow” in a scree plot can be interpreted as salient for that group. An example of a scree plot is presented in Figure 1. Venn diagrams were then used to illustrate term overlap across strata.^32^

## Results

Our final sample included 58 AAB community members from the Philadelphia, Pennsylvania area. One person self-identified as multiracial (AAB and Native American). Table 1 presents the sample demographics. More than two-thirds of the participants were female (*N* = 39, 67.24%). The median age was 51 years with a range of 19–90 years. The age groups were evenly divided for comparison (*N* = 29, 50% in both groups). Additional analyses (presented in text only) were computed for three age groups: <40 years (*n* = 15, 26%), 40–64 years (*n* = 26, 45%), and more than 65 years (*n* – 17, 29%). The sample was highly educated. Twenty-four participants (41.38%) had less than a 4-year college education (*N* = 7 with high school and *N* = 17 with some college.) Thirty-four participants (58.62%) had a 4-year college education or more (*N* = 16 with a 4-year college degree and *N* = 18 with post-graduate education.) Forty-three participants (74.14%) never smoked cigarettes while 15 (25.86%) participants had never smoked cigarettes. Three (5.2%) of participants currently smoked at the time of the interview.

### Free-Listing Results

#### Question 1—Thoughts About People Who Smoke Cigarettes

**Figure 2.**
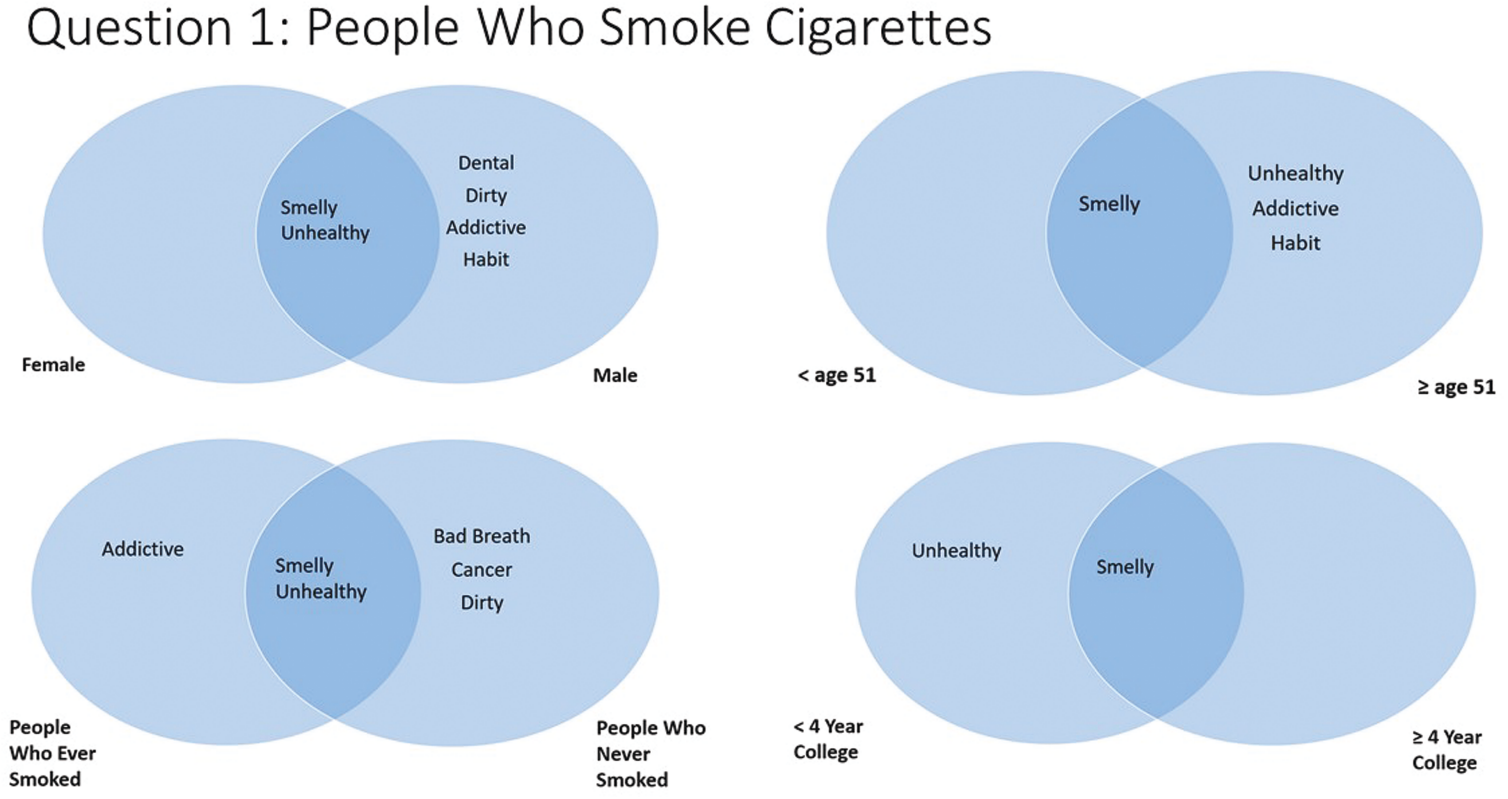
Perceptions about being a person who smokes, by participant demographics.

Scree plots illustrated that the most salient answers for question 1 (thoughts about people who smoke cigarettes) were “smelly” and “unhealthy” (Figure 2). However, males also responded with “dental,” “dirty,” “addictive,” and “habit.” Comparing age groups, individuals atleast 51 years old also included “addictive” and “habit.” In additional age group analyses, for those less than 40 years old, “smelly,” “dental,” and “dirty” were listed. For those ages 40–64, “smelly” and “unhealthy” were listed. For those aged more than 65, “smelly” was the only term listed (not shown in figures). Participants with less than a college degree listed “unhealthy” while more educated participants did not. People who ever smoked also listed “addictive” while people who never smoked listed “bad breath,” “cancer,” and “dirty.”

#### Question 2—Thoughts About Secondhand Smoke

**Figure 3.**
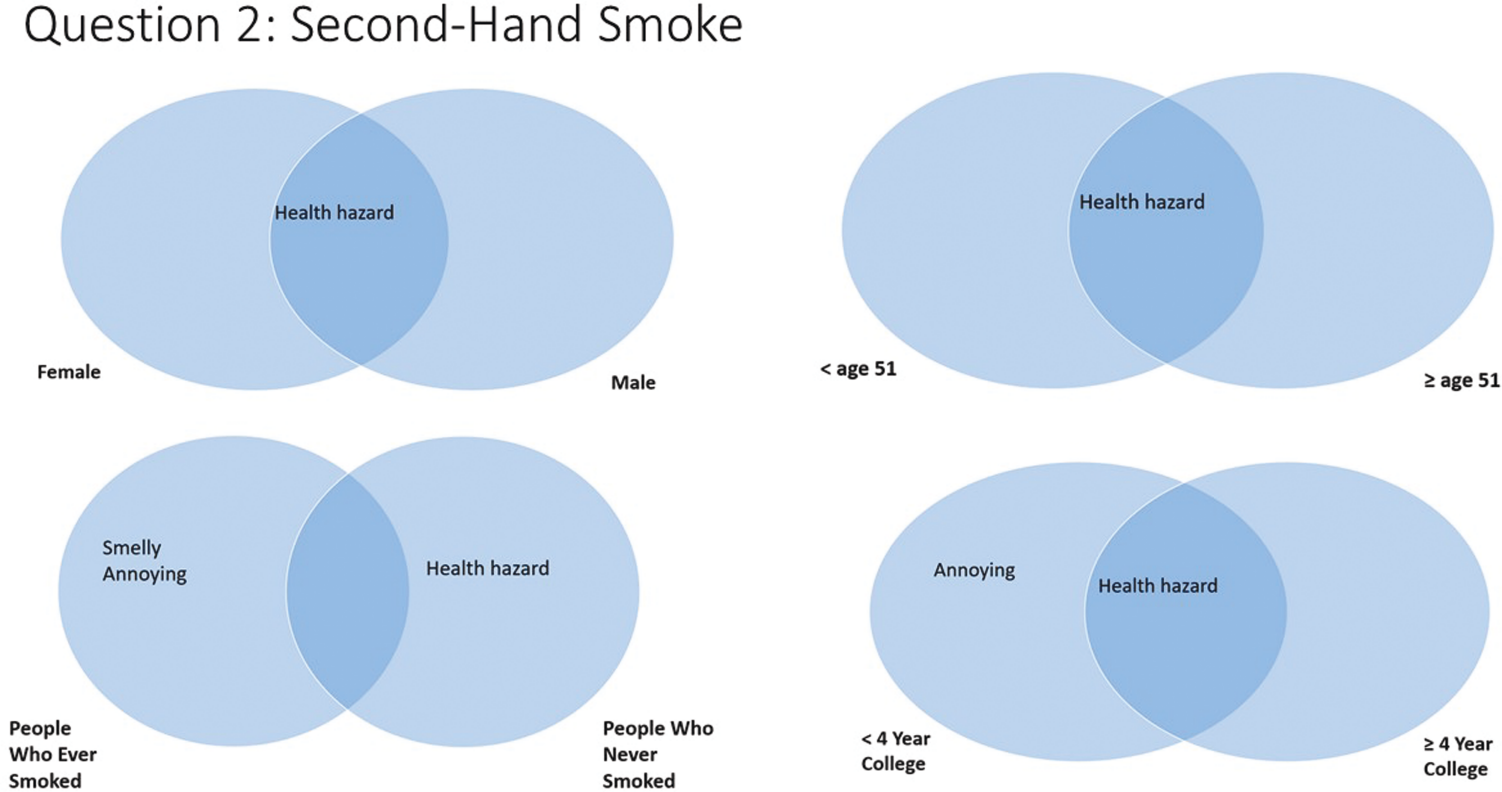
Perceptions about secondhand smoke, by participant demographics.

The most salient answer for question 2 (thoughts about secondhand smoke) was “health hazard,” which was mentioned by every demographic subgroup except people who ever smoked (Figure 3). Those aged more than 65 are also listed “smelly” (not shown in figures). Participants with less than a college degree also listed “annoying.” People who ever smoked listed “smelly” and “annoying.”

#### Question 3—Thoughts About How People Treat People Who Smoke Cigarettes

**Figure 4.**
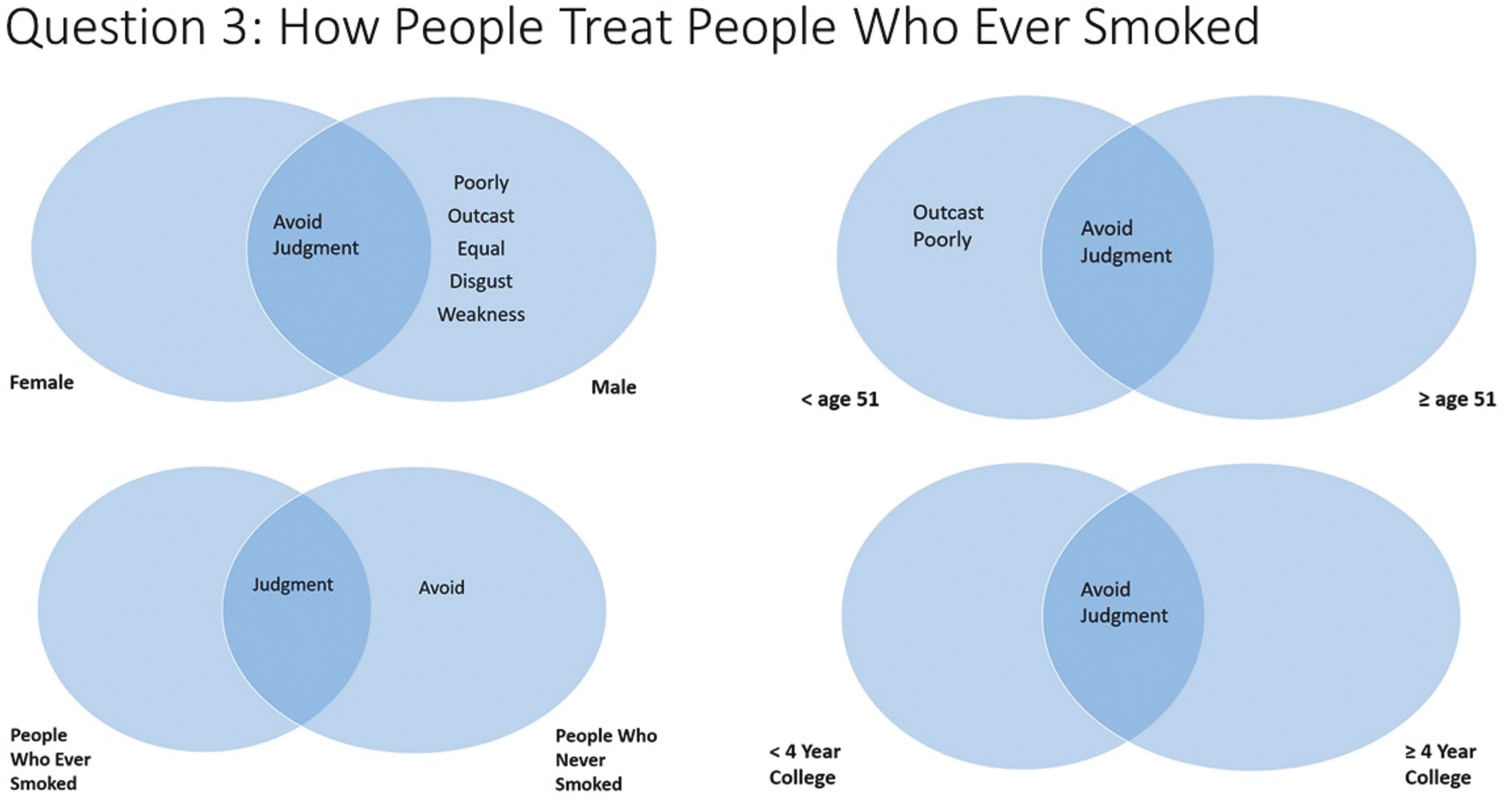
Perceptions about how people who smoke are treated, by participant demographics.

The most salient answers for question 3 (thoughts about how people treat people who smoke cigarettes) were “avoid” and “judgment” (Figure 4). Males and females listed similar responses for question 3. However, males also listed “poorly,” “outcast,” “equal,” “disgust,” and “weakness.” Participants aged less than 51 also listed “outcast” and “poorly.” This was similar to participants aged less than 40 and ages 40–64 (who also mentioned “disgust,” not shown in figures). There were no differences in educational attainment. People who ever smoked listed “judgment” while people who never smoked listed “judgment” and “avoid.”

#### Question 4—Thoughts About Talking to a Doctor About Your Health

**Figure 5.**
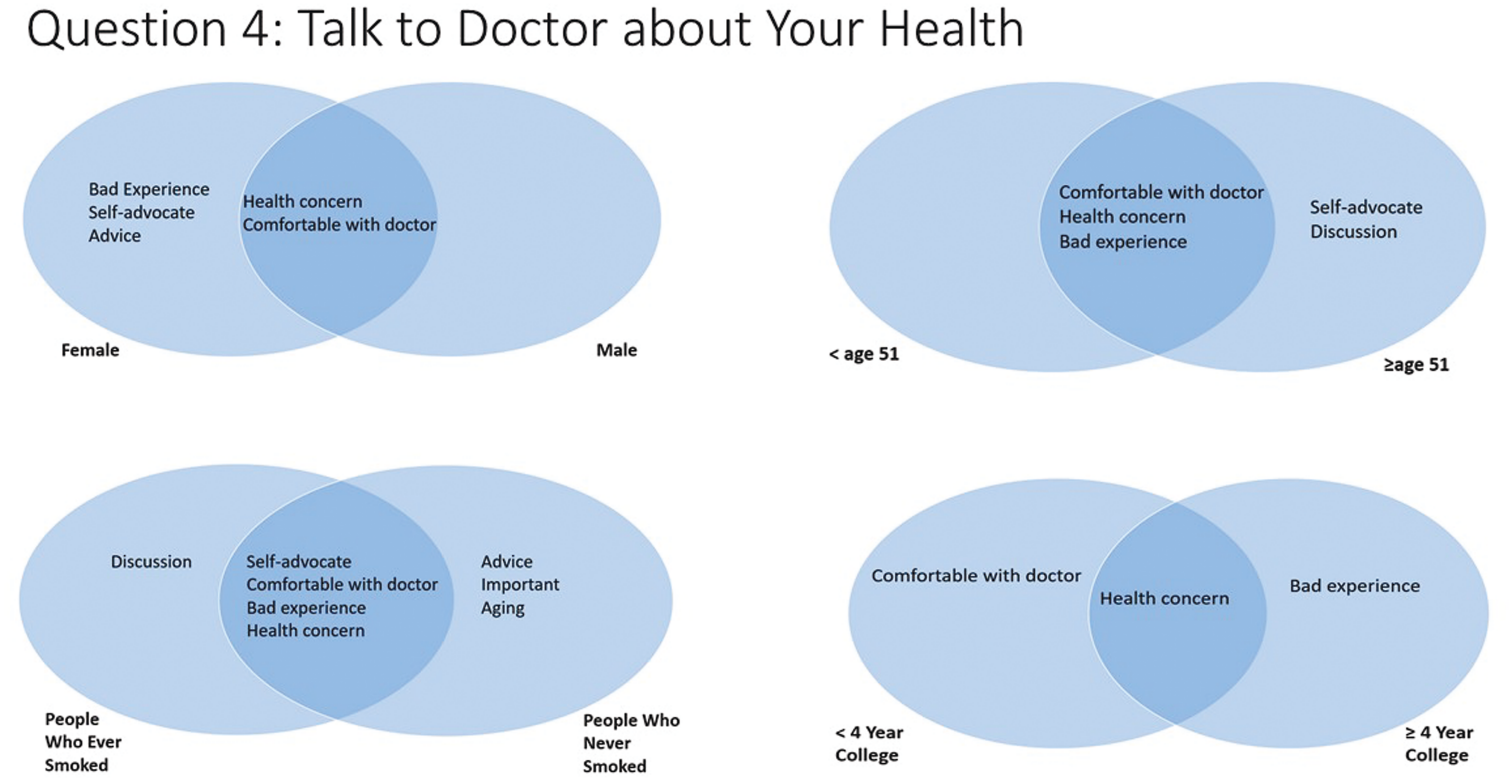
Perceptions about talking to the doctor about health, by participant demographics.

For question 4 (thoughts about talking to a doctor about your health), there were numerous salient responses, including “health concern,” “comfortable with doctor,” “bad experience,” “self-advocate,” “advice,” “important,” “discussion,” “aging,” “treatment,” and “secretive” (Figure 5). Males and females both listed “health concern” and “comfortable with doctor.” However, females also listed “bad experience,” “self-advocate,” and “advice.” Participants aged atleast 51 years listed “self-advocate” and “discussion,” which were not listed by participants less than 51 years. When three age groups were compared, the most salient terms were similar for participants less than 40 years and those 40–65 years old, except that “self-advocate” was salient only among aged 40–64. The only salient term for age more than 65 years was “health concern.” (results not in the figure.) Participants with less than a college degree listed “comfortable with doctor” while more educated participants listed “bad experience.” People who ever smoked additionally listed “discussion.” People who never smoked additionally listed “advice,” “important,” and “aging.”

## Discussion

Our results show that among AAB, smoking is represented by stigmatizing language across gender, age, smoking status, and is primarily associated with negative social interactions, social judgement, and health-related concerns. There were some variations in salient language used across these groups.

When asked about beliefs about a person who smokes, the terms “smelly” and “unhealthy” were the most salient, which clearly indicates negative social interactions, social judgment, and health concerns. The notion that smoking is addictive and habit-forming was mentioned most by older male participants, especially those who have ever smoked. This is an interesting finding as the denormalization of cigarette smoking has been successful in treating cigarette smoking as an addiction.^37^ Those who have ever smoked and older individuals using terms related to addiction indicate that they may hold views that smoking is less of a moral failing. There was very little variation in responses when it came to views on secondhand smoke. The most salient term across all-groups was “health hazard.” Secondhand smoke is well known to be associated with increased risk for heart disease and lung cancer. Its existence allows for the argument that smoking is not just an individual choice, as it can harm others. It is understandable how this could lead to stigmatization of people who smoke as well as self-stigmatization.

When asked about the treatment of individuals who smoke, male participants, and younger participants used the phrases “poorly,” “outcast,” “equal to people who don’t smoke,” “disgust,” and “weakness.” “Judgment” was mentioned by participants regardless of smoking history with people who never smoked also using the phrase “avoid.” These concepts of outcast, disgust, weakness, judgment, and avoidance indicate stigma, either stigmatizing views or the experience of stigma, including self-stigma. Disgust has been previously associated with the moralization of smoking.^4^

The salient terms representing views on discussing health concerns with a health care provider indicated potentially negative healthcare experiences (eg “self-advocate,” “bad experience,” and “secretive.”) As mentioned earlier, negative health care experiences, including the experience of stigma are particularly concerning, as stigma is associated with delays in help-seeking behavior and delays in care for smoking-related conditions such as LC.^19^ LC is a leading cause of cancer-related morbidity and mortality with some of the highest reports of distress and self-stigma among cancer patients.^38–40^ AAB are at increased risk for LC, and AAB men, in particular, are most likely to die from it.^41^ These data suggest that high-risk AAB patients may benefit from effective, stigma-reducing approaches that include social network support for smoking cessation.

The concept of intersectionality between gender and race/ethnicity comes into play in our study. Intersectionality explains how multiple aspects of one’s identity can affect an outcome. In this study, one outcome investigated was one’s potential to access health care, which can change based on the interactions among an individual’s social identities, including race/ethnicity, gender, class, and sexual orientation. This is seen in question four results, where AAB women were more likely to talk about “bad experiences” with health care compared to AAB men. In fact, the term “intersectionality” was coined by the African American feminist and scholar Kimberlé Crenshaw^42^ to point out that African American women have unique experiences and that treating gender and race identities as mutually exclusive is short-sighted. And, when studies have attempted to describe these disparities seen in AAB women based on the factors of sex or race alone, they failed to demonstrate the unique experiences (such as increased risk of death) present in patient subgroups such as AAB women.^43^ Compared to other groups, AAB women are more likely to report differential, biased or discriminatory health care treatment and interactions with health care providers as related to maternal health and reproductive care, chronic illness, and other conditions such as obesity.^44,45^ Prior studies also have shown that AAB women undergo accelerated health decline beginning in their reproductive years.^46^ As a group, they have higher levels of underlying physiological indicators of numerous chronic health conditions^47^ are more likely to be hospitalized^48^ and are more likely to experience poor health outcomes.^44,45^ Interestingly, being a member of two marginalized groups (AAB and women), AAB women also are most likely to report feeling invisible (not heard, not an expert of her own body, disregarded preferences, and in need of more self-advocacy) in a variety of health care settings.^45,49,50^ Moreover, implicit bias from health care providers also has been associated with disparities in treatment and health outcomes.^51^ Therefore, it is reasonable to assume that these issues may be found in the primary care setting. Further research into how intersectionality affects smoking-related stigma and resultant access to care and interactions with providers should be investigated.

A large majority of participants in this study self-disclosed that they did not smoke. A low smoking rate among this convenience sample may partially be attributable to what Stuber et al.^25^ assert as the rise in the “social unacceptability of smoking” which has contributed to significant decreases in the use of tobacco products. Among adults racialized as AAB, cigarette smoking has substantially decreased over the last 40 years.^52^ Moreover, research has reported that the prevalence of smoking is lowest among persons who have earned a college degree or higher.^53,54^ Given that more than half of the respondents in this sample fit this description and the use of snowball recruitment, having a sample that has a large percentage of nonsmoking behavior is not necessarily surprising. Although the study does not have an abundance of perspectives from people who smoke, demographic shifts in smoking behaviors suggest that exploration of smoking stigma as viewed by people with diverse smoking histories is worthwhile.

## Limitations

One limitation of this study is that using the snowball approach (convenience sampling) may have resulted in a sample that is on average more educated than the general AAB population. This approach is useful for recruiting from among groups that are considered hard-to-reach, such as communities that have historically experienced substantial social marginalization.^55^ However, the findings in this study cannot be generalized to a larger population, particularly outside of the Philadelphia region. We acknowledge this in the interpretation of our study results and suggest that these findings may be more representative of a relevant and potentially influential subset of the AAB population in the Philadelphia metropolitan region. Nonetheless, the results provide a baseline of data useful for further investigation that considers if homogenous views about smoking stigma exist among this population. Future studies also will utilize a broader geographic sample.

Additionally, our study included only 15 people who smoked cigarettes, and therefore comparisons across groups are somewhat limited. Although Keddem et al.^55^ recommends “aiming for at least 20 interviews per group” to make cross-comparisons, we moved forward with our comparison of responses between 39 females and 19 men. The authors did not specifically address if one less person (one man) of the minimum number recommended substantially alters saturation. However, in light of this difference, we found the data to be useful and acknowledged that the findings should be considered cautiously.

Finally, Keddem et al.^32^ assert one limitation of free listing is the lack of depth obtained from single-word or short-phrase responses from study participants when compared to data collected from semi-structured interviews or focus groups. Although responses such as “smelly” or “unhealthy” may reflect some respondents’ thoughts about cigarette smoking, it does not capture the range or context concerning these concepts. For instance, how much of a smell associated with cigarette smoking is considered “smelly?” Does “unhealthy” refer only to the act of cigarette smoking or does it include additional factors? Additionally, the authors state that bias may be introduced during the data cleaning stage if a less than rigorous process is performed, an inductive approach is not undertaken, or multiple perspectives are not considered. However, we attempted to mitigate researcher bias by using the respondents’ own words.

## Conclusions

Stigma has been identified as a primary barrier to health care for LC patients, but there is little known about how stigma factors into LC screening and the social support AAB patients receive. Generally speaking, people who smoke and experience higher levels of stigma have more quiet attempts but may be less likely to seek help quitting.^56^ Given this, the goal of this study was to examine the salience of smoking-related stigmas among AAB demographic groups. Future studies are needed to explore how smoking-related stigmas impact patient adherence to smoking cessation programs and LC screening protocols among vulnerable populations. We will also explore the role of self-efficacy, health literacy, and social support in lessening stigma and smoking behavior and improving the utilization of screening services for eligible patients.

## Data Availability

The datasets analyzed during the current study are available from the corresponding author upon reasonable request.
